# Evidence for an ACE2-Independent Entry Pathway That Can Protect from Neutralization by an Antibody Used for COVID-19 Therapy

**DOI:** 10.1128/mbio.00364-22

**Published:** 2022-04-25

**Authors:** Markus Hoffmann, Anzhalika Sidarovich, Prerna Arora, Nadine Krüger, Inga Nehlmeier, Amy Kempf, Luise Graichen, Martin S. Winkler, Daniela Niemeyer, Christine Goffinet, Christian Drosten, Sebastian Schulz, Hans-Martin Jäck, Stefan Pöhlmann

**Affiliations:** a Infection Biology Unit, German Primate Center, Göttingen, Germany; b Faculty of Biology and Psychology, Georg-August-University Göttingen, Göttingen, Germany; c Department of Anesthesiology, University of Göttingen Medical Center, and Georg-August University of Göttingen, Göttingen, Germany; d Institute of Virology, Campus Charité Mitte, Charité-Universitätsmedizin Berlin, Berlin, Germany; e German Centre for Infection Research, associated partner Charité, Berlin, Germany; f Division of Molecular Immunology, Department of Internal Medicine 3, Friedrich-Alexander University of Erlangen-Nürnberg, Erlangen, Germany; Columbia University/HHMI

**Keywords:** ACE2, COVID-19, antibody, neutralization, spike

## Abstract

SARS-CoV-2 variants of concern (VOC) acquired mutations in the spike (S) protein, including E484K, that confer resistance to neutralizing antibodies. However, it is incompletely understood how these mutations impact viral entry into host cells. Here, we analyzed how mutations at position 484 that have been detected in COVID-19 patients impact cell entry and antibody-mediated neutralization. We report that mutation E484D markedly increased SARS-CoV-2 S-driven entry into the hepatoma cell line Huh-7 and the lung cell NCI-H1299 without augmenting ACE2 binding. Notably, mutation E484D largely rescued Huh-7 but not Vero cell entry from blockade by the neutralizing antibody Imdevimab and rendered Huh-7 cell entry ACE2-independent. These results suggest that the naturally occurring mutation E484D allows SARS-CoV-2 to employ an ACE2-independent mechanism for entry that is largely insensitive against Imdevimab, an antibody employed for COVID-19 therapy.

## OBSERVATION

**FIG 1:**
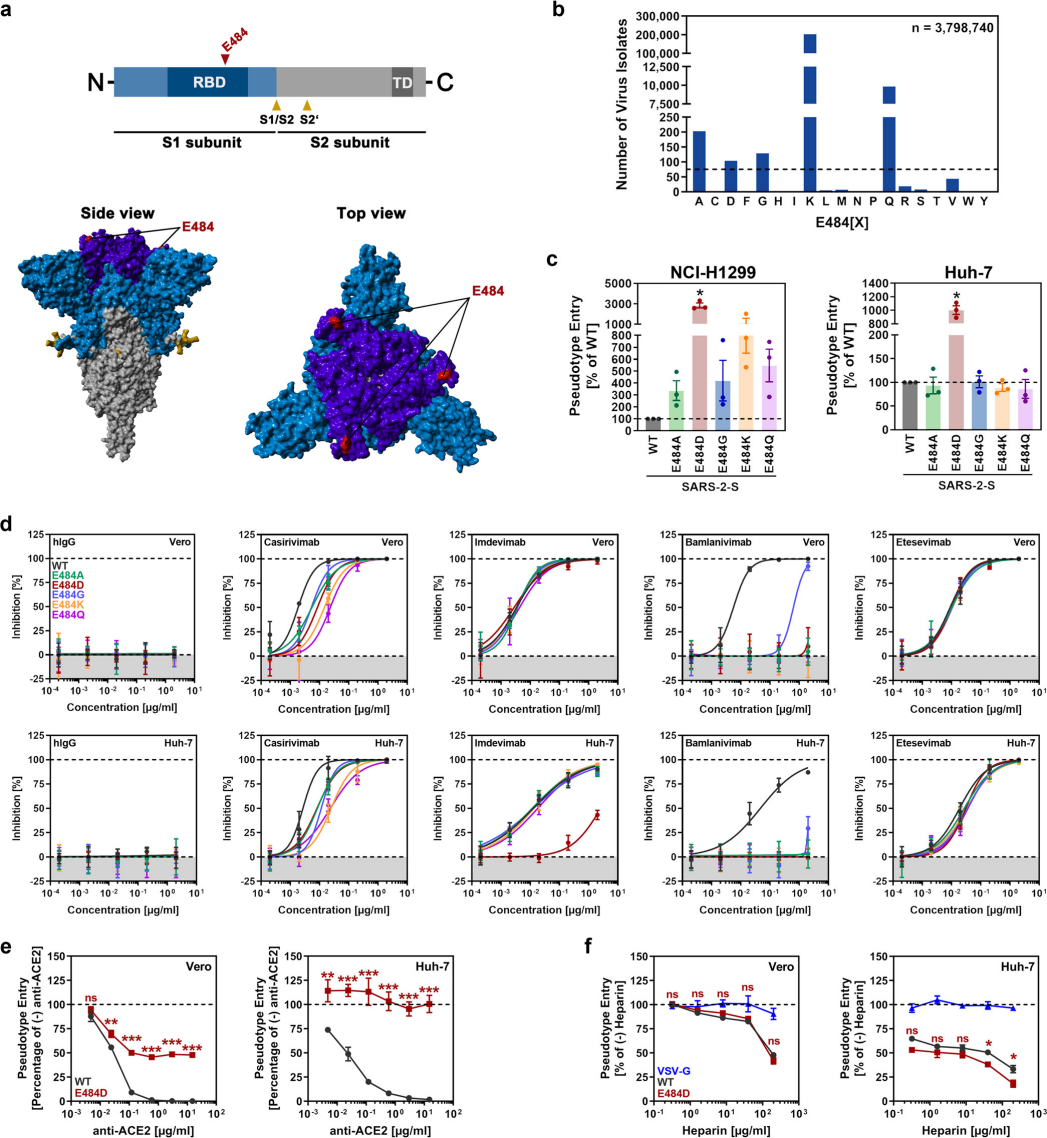
Spike mutation E484D leads to cell line-dependent enhancement of infection in a potentially ACE2-independent manner and allows escape from neutralization by Imdevimab. (a) Spike (S) protein scheme (abbreviations: RBD = receptor binding domain, TD = transmembrane domain) and location of residue E484 in the context of the three-dimensional S protein structure (color code: Light blue = S1 subunit [non-RBD], dark blue = RBD, gray = S2 subunit, red = residue E484). (b) Frequency of mutations at S protein residue E484 (letters indicate amino acid exchanges, single letter code). The dashed line shows the threshold for selection of mutants for in-depth analysis (minimum frequency = 75 entries in the GISAID database as of 29.09.2021). (c) Mutations at position E484 lead to cell line-dependent augmentation of infection. Particles pseudotyped with the indicated S proteins were inoculated onto H1299 (human, lung) and Huh-7 (human, liver) cells. At 16–18h postinoculation, transduction efficiency was analyzed by measuring virus-encoded luciferase activity in cell lysates. Presented are the average (mean) data from three biological replicates (each conducted with four technical replicates), for which transduction was normalized against wild-type (WT) SARS-CoV-2 S (set as 1). Error bars indicate the standard error of the mean (SEM). (d) Mutation E484D enables evasion from Imdevimab-mediated neutralization in Huh-7 but not Vero cells. Particles pseudotyped with the indicated S proteins were preincubated (30 min, 37°C) with different concentrations of monoclonal antibodies used for COVID-19 therapy (Casirivimab, Imdevimab, Bamlanivimab, Etesevimab) or an unrelated control antibody (hIgG), before being inoculated onto Vero and Huh-7 cells. Transduction efficiency was quantified at 16–18h postinoculation as described for panel c and normalized against samples that did not contain antibody (= 0% inhibition). Presented are the average (mean) data from a single experiment conducted with four technical replicates. Results were confirmed in a separate experiment. Error bars indicate the standard deviation (SD). (e) Evidence that mutation E484D allows for ACE2-independet cell entry. Vero and Huh-7 were preincubated (30 min, 37°C) with different concentrations of anti-ACE2 antibody, before particles pseudotyped with the indicated S proteins were added on top. Transduction efficiency was quantified at 16–18h postinoculation as described for panel c of Fig. 1. Presented are the average (mean) data from three biological replicates (each conducted with four technical replicates), for which transduction was normalized against samples that did not contain antibody (= 100% pseudotype entry). Error bars indicate the SEM. (f) S protein-driven entry into Huh-7 cells depends on heparan sulfate. Particles pseudotyped with the indicated S proteins (or VSV-G) were preincubated (30 min, 37°C) with different concentrations of heparin before being inoculated on to Vero and Huh-7 cells. Transduction efficiency was quantified at 16–18h postinoculation as described for panel c of Fig. 1. Presented are the average (mean) data from three biological replicates (each conducted with four technical replicates), for which transduction was normalized against samples that did not contain heparin (= 100% pseudotype entry). Error bars indicate the SEM. Statistical analysis: For panel c, statistical significance was assessed by two-tailed Student's *t* test with Welch’s correction, whereas for panels e and f, statistical significance was assessed by two-way analysis of variance (ANOVA) with Sidak’s *post hoc* test (*P* > 0.05, not significant [ns; not indicated in panel c]; *P* ≤ 0.05, *; *P* ≤ 0.01, **; *P* ≤ 0.001, ***).

The spike (S) protein of SARS-CoV-2 mediates entry into host cells and is the key target for neutralizing antibodies induced upon infection and vaccination or used for COVID-19 therapy (1). The S proteins of SARS-CoV-2 variants of concern (VOC) harbor mutations that reduce susceptibility to antibody-mediated neutralization and may alter virus-host cell interactions (2). Amino acid residue E484 is located in the receptor binding domain (RBD) of the S protein (Fig. 1a), which binds to the cellular receptor ACE2, and VOCs Beta (B.1.351) and Gamma (P.1), harbor mutation E484K, which has been associated with neutralization resistance (2, 3). However, a systematic comparison of the role of amino acid residue 484 in antibody-mediated neutralization and host cell entry is so far lacking.

## E484D INCREASES ENTRY INTO HUH-7 CELLS

We employed rhabdoviral pseudotypes, which faithfully mimic SARS-CoV-2 entry into cells and its inhibition by antibodies (4), to analyze the role of amino acid residue 484 in host cell entry and its inhibition (see material and methods in the supplement). The analysis of SARS-CoV-2 sequences deposited in GISAID (as of 29.09.2021) revealed that the most frequent substitution at position 484 is E484K, followed E484Q, E484A, E484D and E484G (Fig. 1b). We introduced these substitutions into the S protein of B.1 (which is identical to the S protein of the Wuhan-01 isolate except for mutation D614G) and analyzed entry into a diverse panel of cell lines. None of the mutations appreciably altered binding to soluble ACE2 (Fig. S1a). The mutations did not enhance entry into 293T, BEAS-2B, Caco-2, Calu-3, and Vero cells but some mutations caused a moderate decrease in cellular entry (Fig. S1b). In contrast, mutation E484D moderately increased entry into A549, HOS and NCI-H727 cells (Fig. S1c) and markedly augmented entry into NCI-H1299 and Huh-7 cells (Fig. 1c and Fig. S1d). Further, mutations E484Q, E484A, E484G and particularly E484K also increased entry into NCI-H1299 cells (Fig. 1c), indicating that amino acid residue 484 may modulate viral cell tropism.

10.1128/mbio.00364-22.2 FIG S1 Cell line-dependent enhancement of infection by mutations at residue E484. (a) 293T cells transiently expressing the indicated S proteins (or no S protein, control) upon transfection were successively incubated with soluble human ACE2 containing a C-terminal Fc-tag (derived from human IgG) and AlexaFluor488-conjugated anti-human antibody before S protein binding to ACE2 was analyzed by flow cytometry. For this, the geometric mean channel fluorescence at 488 nm was measured. Presented are the average (mean) data from three biological replicates (each conducted with single samples). Error bars indicate the SD. (b) Particles pseudotyped with the indicated S proteins were inoculated onto 293T (human, kidney), BEAS-2B (human, lung), Caco-2 (human, intestine), Calu-3 (human, lung) and Vero (African green monkey, kidney) cells. Quantification of transduction efficiency and data normalization were performed as described for panel c of fig. 1. Presented are the average (mean) data from three biological replicates (each conducted with four technical replicates). Error bars indicate the SEM. (c) Particles pseudotyped with the indicated S proteins were inoculated onto A549 (human, lung), HOS (human, bone) and NCI-H727 (human, lung) cells. Quantification of transduction efficiency and data normalization were performed as described for panel c of fig. 1. Presented are the average (mean) data from three biological replicates (each conducted with four technical replicates). Error bars indicate the SEM. (d) The experiment was performed as described in the legend of figure 1c. Presented are the average (mean) data from the same biological replicates as presented in figure 1c and figure S1b and c, with the difference that this time transduction was normalized against signals obtained from cells inoculated with particles bearing no viral glycoprotein (background, set as 1). Further, transduction data of particles bearing VSV-G were included. Error bars indicate the SEM. Statistical analysis: For panels a-c, statistical significance was assessed by two-tailed Student’s t-test with Welch’s correction (*P* > 0.05, not significant [ns; not indicated in panels b and c]; *P* ≤ 0.05, *; *P* ≤ 0.01, **; *P* ≤ 0.001, ***). Download FIG S1, TIF file, 2.3 MB. Copyright © 2022 Hoffmann et al. 2022 Hoffmann et al. https://creativecommons.org/licenses/by/4.0/ This content is distributed under the terms of the Creative Commons Attribution 4.0 International license.

## E484D RENDERS HUH-7 CELL ENTRY LESS SENSITIVE TO IMDEVIMAB

We next hypothesized that the increased Huh-7 cell entry of mutant E484D might reflect altered interactions with entry factors which, in turn, might be associated with altered neutralization by antibodies. To address this possibility, we compared neutralization using Vero (which allowed comparable entry of all mutants tested) and Huh-7 cells (which allowed for increased entry of mutant E484D) as target cells. Neutralization by convalescent plasma revealed that mutation E484K, which is present in VOCs Beta and Gamma, and mutation E484Q, found in the variant of interest Kappa (B.1.617.1), markedly reduced neutralization sensitivity, in keeping with published data (2, 3), while the other mutations had minor or no effects (Fig. S2a-b). No appreciable differences were observed when Vero or Huh-7 cells were used as targets (Fig. S2a-b). We next analyzed neutralization by antibodies employed for COVID-19 therapy. All mutations studied were compatible with neutralization by Casirivimab, although minor differences in neutralization sensitivity were noted, and Etesevimab (Fig. 1d). In contrast, all mutations studied reduced or abrogated neutralization by Bamlanivimab, as expected, since E484 resides in the center of the epitope recognized by this antibody (Fig. S2c), and the results were independent of the target cell line used (Fig. 1d). Surprisingly, exchange E484D protected from Imdevimab-mediated neutralization in Huh-7 but not Vero cells (Fig. 1d), although the antibody epitope is located distantly from the mutated residue (Fig. S2c) and Imdevimab binding to the S protein was not compromised by mutation E484D (Fig. S2d). This finding suggests that mutant E484D may utilize an altered strategy for entry into Huh-7 cells that is associated with reduced sensitivity to neutralization by certain antibodies.

10.1128/mbio.00364-22.3 FIG S2 Mutations at S protein residue E484 modulate sensitivity to antibody-mediated neutralization by convalescent plasma in a cell line-independent manner. (a) Particles pseudotyped with the indicated S proteins were preincubated (30 min, 37°C) with different dilutions of convalescent plasma, before being inoculated onto Vero and Huh-7 cells. Transduction efficiency was quantified at 16–18 h postinoculation as described for panel d of fig. 1 and used to calculate the plasma dilution factor that leads to 50% reduction in S protein-driven cell entry (neutralizing titer 50, NT50, upper panels) and the fold change in neutralization sensitivity between WT and mutant S proteins (lower panels). Presented are the data from a total of eight convalescent plasma (boxes indicate 25–75% quartiles, horizontal and vertical lines indicate the median and range, respectively). (b) The unprocessed (upper panel) and processed (Fold change between NT50 values obtained for Vero versus Huh-7 cells, lower panel) NT50 data presented in panel a were graphed for comparison of neutralization of S protein-driven entry by convalescent plasma on Vero (gray) versus Huh-7 (red) cells. Presented are the data from a total of eight convalescent plasma (boxes indicate 25–75% quartiles, horizontal and vertical lines indicate the median and range, respectively). (c) Model of the SARS-CoV-2 S RBD (gray) in which residues that directly interact with ACE2 (green) or form the epitope that is bound by monoclonal antibodies used for COVID-19 therapy (blue) are highlighted (residue E484 is marked in red). (d) Plasmids encoding SARS-CoV-2 WT and mutant E484D were transiently transfected into 293T cells and binding of the indicated antibodies to S protein on the cell surface was analyzed by flow cytometry using an Alexa Fluor-488-conjugated anti-human secondary antibody. Presented are the average (mean) data from three biological replicates (each conducted with single samples) showing either the geometric mean channel fluorescence at 488 nm (left panel), or the relative change in antibody binding between WT and mutants SARS-CoV-2 S (right panel). Error bars indicate the SD. Statistical analysis: For panels A and B, statistical significance was assessed by two-tailed Mann-Whitney test, while for panel D, statistical significance was assessed by two-tailed Student’s t-test with Welch’s correction[ns]; *P* ≤ 0.05, *; *P* ≤ 0.01, **; *P* ≤ 0.001, ***). Download FIG S2, TIF file, 2.9 MB. Copyright © 2022 Hoffmann et al. 2022 Hoffmann et al. https://creativecommons.org/licenses/by/4.0/ This content is distributed under the terms of the Creative Commons Attribution 4.0 International license.

## E484D ALLOWS FOR ACE2-INDEPENDENT ENTRY INTO HUH-7 CELLS

We next thought to gain initial insights into how mutant E484D enters Huh-7 cells. We found that soluble ACE2 robustly and comparably blocked Vero and Huh-7 cell entry of WT and mutant E484D (Fig. S3a). In contrast, mutant E484D was largely resistant against inhibition by two antibodies raised against the ACE2 ectodomain (Fig. 1e and Fig. S3b). Specifically, the antibodies efficiently blocked WT entry into Vero and Huh-7 cells but exerted little (Vero) or no (Huh-7) inhibitory activity against mutant E484D (Fig. 1e and Fig. S3b). Thus, mutation E484D might either markedly alter S protein interactions with ACE2, rendering entry resistant to the ACE2-antibodies employed in the present study, or might allow for ACE2-independent entry, as demonstrated by a separate study focusing on the cell line H522 (5), or both. Next, we asked whether engagement of heparan sulfate proteoglycans, which can augment SARS-CoV-2 infection (6, 7), promoted entry into Huh-7 cells. Vero cells were used as reference. Preincubation of S protein-bearing particles with soluble heparin moderately and comparably reduced WT and E484D entry into Vero cells (Fig. 1f). Inhibition of entry was more pronounced in Huh-7 cells and mutant E484D was slightly more efficiently inhibited compared to WT (Fig. 1f). Thus, heparan sulfate-containing proteoglycans might partially account for the increased Huh-7 cell entry of E484D, as suggested by a separate study for H522 cells (5) but are unlikely to be the only explanation.

10.1128/mbio.00364-22.4 FIG S3 Soluble ACE2 inhibits entry driven by SARS-CoV-2 S WT and mutant E484D with similar efficiency while anti-ACE2 antibody fails to inhibit Huh-7 cell entry driven by mutant E484D. (a) Particles pseudotyped with the indicated S proteins were preincubated (30 min, 37°C) with different dilutions of soluble ACE2 before being inoculated on Vero and Huh-7 cells. Transduction efficiency was quantified at 16–18 h postinoculation as described for panel e of fig. 1. Presented are the average (mean) data from three biological replicates (each conducted with four technical replicates), for which transduction was normalized against samples that did not contain soluble ACE2 (= 100% pseudotype entry). Error bars indicate the SEM. (b) Inhibition of SARS-CoV-2 S WT and mutant E484D-mediated entry into Vero and Huh-7 cells by an anti-ACE2 antibody. The experiment was conducted as described in the legend to figure 1e but anti-ACE2 antibody 10108-MM36 was used. Presented are the average (mean) data from three biological replicates (each conducted with four technical replicates), for which transduction was normalized against samples that did not contain anti-ACE2 antibody (= 100% pseudotype entry). Error bars indicate the SEM. Statistical analysis: Statistical significance was assessed by two-way ANOVA with Sidak’s post-hoc test (*P* > 0.05, not significant [ns]; *P* ≤ 0.05, *; *P* ≤ 0.01, **; *P* ≤ 0.001, ***). Download FIG S3, TIF file, 0.9 MB. Copyright © 2022 Hoffmann et al. 2022 Hoffmann et al. https://creativecommons.org/licenses/by/4.0/ This content is distributed under the terms of the Creative Commons Attribution 4.0 International license.

## DISCUSSION

As of 29.09.2021, mutation E484D has been reported globally in a total of 105 out of 3,798,740 SARS-CoV-2 sequences deposited in the GISAID (Global Initiative on Sharing All Influenza Data) database with increasing frequency in 2021 and is associated with multiple SARS-CoV-2 lineages (Fig. S4a-c). Our results show that mutation E484D increases entry into Huh-7 cells, which is associated with altered or, perhaps more likely, abrogated ACE2 usage and Imdevimab resistance. These findings await confirmation with authentic SARS-CoV-2 but are in keeping with a previous study showing that mutation E484D allowed ACE2-independent entry of authentic SARS-CoV-2 into the lung cell line H522, as demonstrated by ACE2 expression (not detected in H522 cells), antibody inhibition and ACE2 knockout analyses (5). Further, the study demonstrated that entry into H522 cells, although being ACE2-independent, was highly susceptible to blockade by soluble ACE2 and antibodies directed against the RBD and the N-terminal domain (NTD) of the S protein (5). The presence of an ACE2-independent pathway operative in certain cell lines raises interesting questions mainly regarding the nature of the cellular factor(s) that allow S protein-mediated viral attachment to target cells but also regarding the enzymes exploited for proteolytic activation of the S protein. The present study and previous work (5) suggest that mutant E484D is activated by the endolysosomal protease cathepsin L (Fig. S5a) and exploits HSPG but not neuropilin-1 for ACE2-independent entry. However, HSPG usage is unlikely to be solely responsible for the ACE2-independent Huh-7 cell entry of mutant E484D, considering that entry inhibition by heparin was incomplete. The cellular lectin ASGR1 is expressed by Huh-7 cells and has recently been reported to serve as alternative SARS-CoV-2 receptor (8). Therefore, future studies should examine whether ASGR1 contributes to Huh-7 cell entry of mutant E484D. Another receptor candidate to be considered is activated integrin α5β1, which was also recently shown to allow for ACE2-independent entry (9).

10.1128/mbio.00364-22.5 FIG S4 S protein mutation E484D is globally distributed and found in several SARS-CoV-2 lineages. (a) Frequency of SARS-CoV-2 sequences that harbor S protein mutation E484D with respect to their global (per continent) distribution (based on sequences deposited in the GISAID database, *n* = 105). (b) Frequency of SARS-CoV-2 sequences that harbor S protein mutation E484D with respect to the time (month/year) of sampling (based on sequences deposited in the GISAID database, *n* = 105). Bars indicate the number of isolates per month while the red line indicates cumulative numbers. (c) Frequency of SARS-CoV-2 sequences that harbor S protein mutation E484D with respect to the SARS-CoV-2 PANGO lineages (based on sequences deposited in the GISAID database, *n* = 105). Download FIG S4, TIF file, 0.8 MB. Copyright © 2022 Hoffmann et al. 2022 Hoffmann et al. https://creativecommons.org/licenses/by/4.0/ This content is distributed under the terms of the Creative Commons Attribution 4.0 International license.

10.1128/mbio.00364-22.6 FIG S5 S protein mutant E484D facilitates cathepsin B/L-dependent entry. (a) Vero and Huh-7 cells were preincubated with the indicated inhibitors for 1 h and then transduced with VSV reporter particles bearing SARS-CoV-2 WT spike or mutant E484D. Luciferase activity in cell lysates was determined at 16 h posttransduction. Presented are the average (mean) data from three biological replicates (each conducted with four technical replicates), for which transduction was normalized against samples that did not receive inhibitor (= 100% pseudotype entry). Error bars indicate the SEM. (b) The experiment was conducted as described for panel a but cells were preincubated with amphotericin B which rescues viral entry from blockade by the cellular restriction factors IFITM2 and IFITM3. Presented are the average (mean) data from three biological replicates (each conducted with four technical replicates), for which transduction was normalized against samples that did not receive amphotericin B (pseudotype entry = 1). Error bars indicate the SEM. (c) Particles pseudotyped with the indicated S proteins were inoculated onto H1299 (human, lung) cells. Quantification of transduction efficiency and data normalization were performed as described for panel c of fig. 1. Presented are the average (mean) data from three biological replicates (each conducted with four technical replicates). Error bars indicate the SEM. Statistical analysis: For panel a, statistical significance was assessed by two-way ANOVA with Sidak’s post-hoc test, while for panels b and c, statistical significance was assessed by two-tailed Student’s t-test with Welch’s correction (*P* > 0.05, not significant [ns]; *P* ≤ 0.05, *; *P* ≤ 0.01, **; *P* ≤ 0.001, ***). Download FIG S5, TIF file, 1.7 MB. Copyright © 2022 Hoffmann et al. 2022 Hoffmann et al. https://creativecommons.org/licenses/by/4.0/ This content is distributed under the terms of the Creative Commons Attribution 4.0 International license.

Mutation E484D did not interfere with binding of the antibody Imdevimab to the S protein but selectively conferred resistance to neutralization by Imdevimab in Huh-7 cells, suggesting that the Imdevimab epitope is dispensable for use of the postulated alternative (i.e., ACE2-independent) entry route. In contrast, Casirivimab and Etesevimab neutralized both Vero and Huh-7 cell entry with high efficiency, indicating that the epitopes recognized by Casirivimab and Etesevimab are required for entry via both the “conventional” (ACE2-dependent) and the alternative (ACE2-independent) route. Whether the cell type-dependent effect of E484D on neutralization sensitivity has implications for viral control by antibodies in the host is unknown. The notion that E484D can emerge upon adaptation of SARS-CoV to growth in cell culture (10) and may increase sensitivity toward the SARS-CoV-2 restriction factors IFITM2/IFITM3 (11, 12), as determined by augmentation of entry upon amphotericin B treatment (Fig. S5b), argues against an important role *in vivo*. On the other hand, mutation E484D (13) and SARS-CoV-2 infection of liver (14) has been detected in COVID-19 patients and ACE2-independent infection of liver cells might contribute to viral pathogenesis. Finally, it is noteworthy that several substitutions of residue E484, including the frequently occurring E484K present in VOCs Beta and Gamma, increased entry into the lung cell line NCI-H1299, which allowed for augmented entry of particles bearing S proteins of VOC compared to WT S protein (Fig. S5c). Thus, analysis of the mechanisms underlying increased VOC entry into H1299 cells could yield valuable insights into why VOC outcompete previously circulating viruses.

10.1128/mbio.00364-22.1 TEXT S1 Materials and Methods. Download Text S1, DOCX file, 0.05 MB. Copyright © 2022 Hoffmann et al. 2022 Hoffmann et al. https://creativecommons.org/licenses/by/4.0/ This content is distributed under the terms of the Creative Commons Attribution 4.0 International license.
