# Supplementary figures and images for: Evidence for an ACE2-Independent Entry Pathway That Can Protect from Neutralization by an Antibody Used for COVID-19 Therapy
Source: mBio. 2022 Apr 25;13(3):e00364-22. doi: 10.1128/mbio.00364-22 (PMC9239067; doi:10.1128/mbio.00364-22)

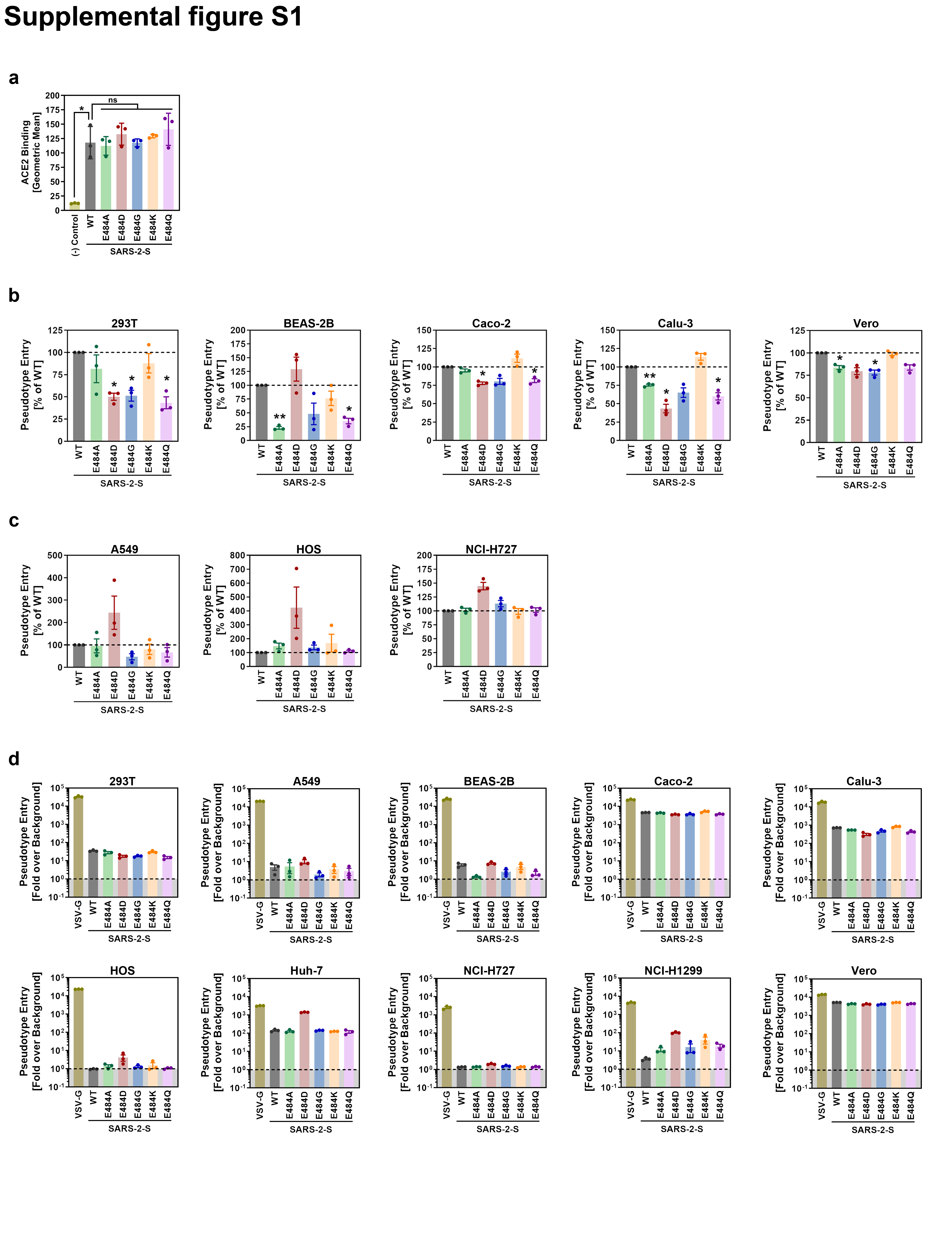

Supplement: FIG S1 [file mbio.00364-22-sf001.tif]

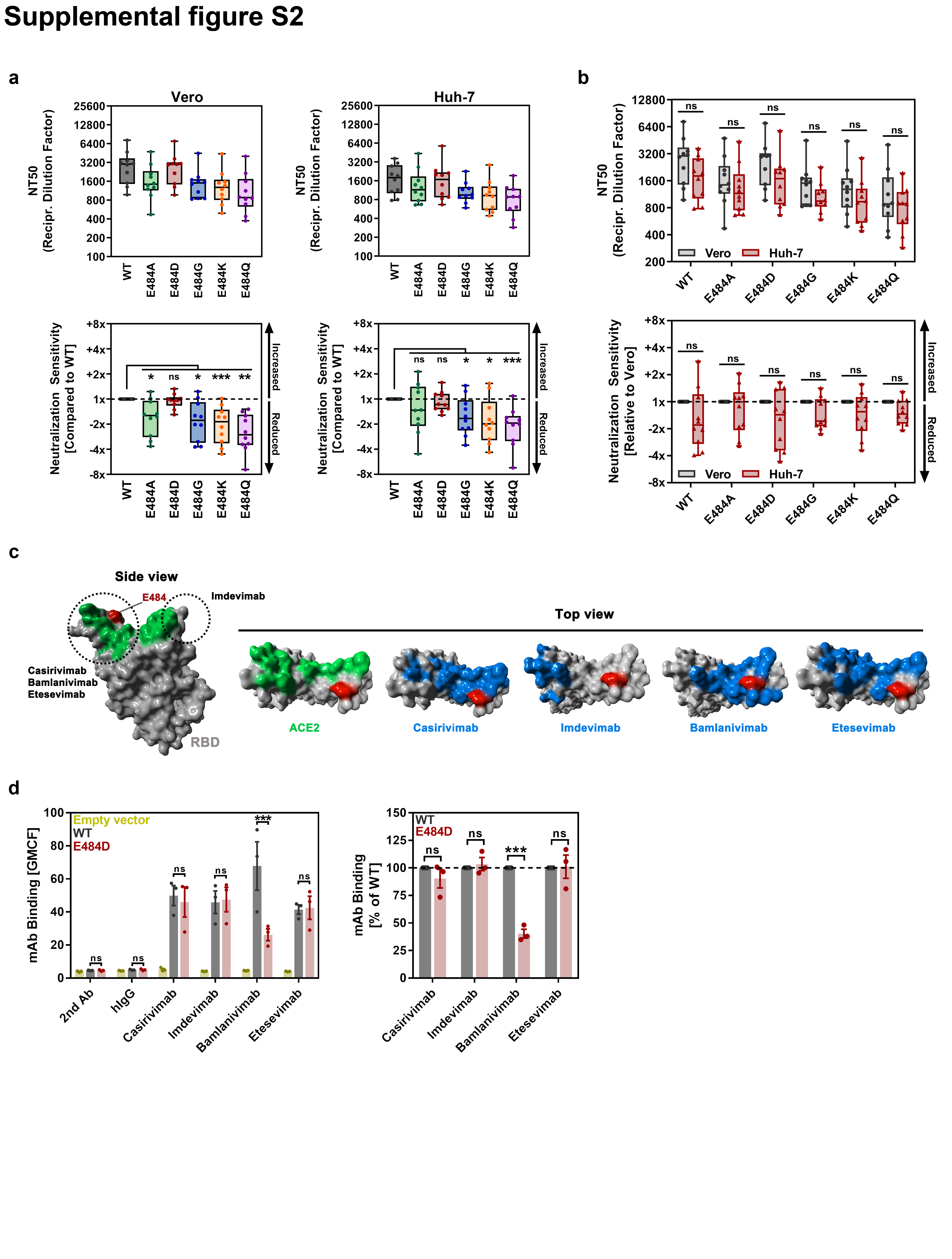

Supplement: FIG S2 [file mbio.00364-22-sf002.tif]

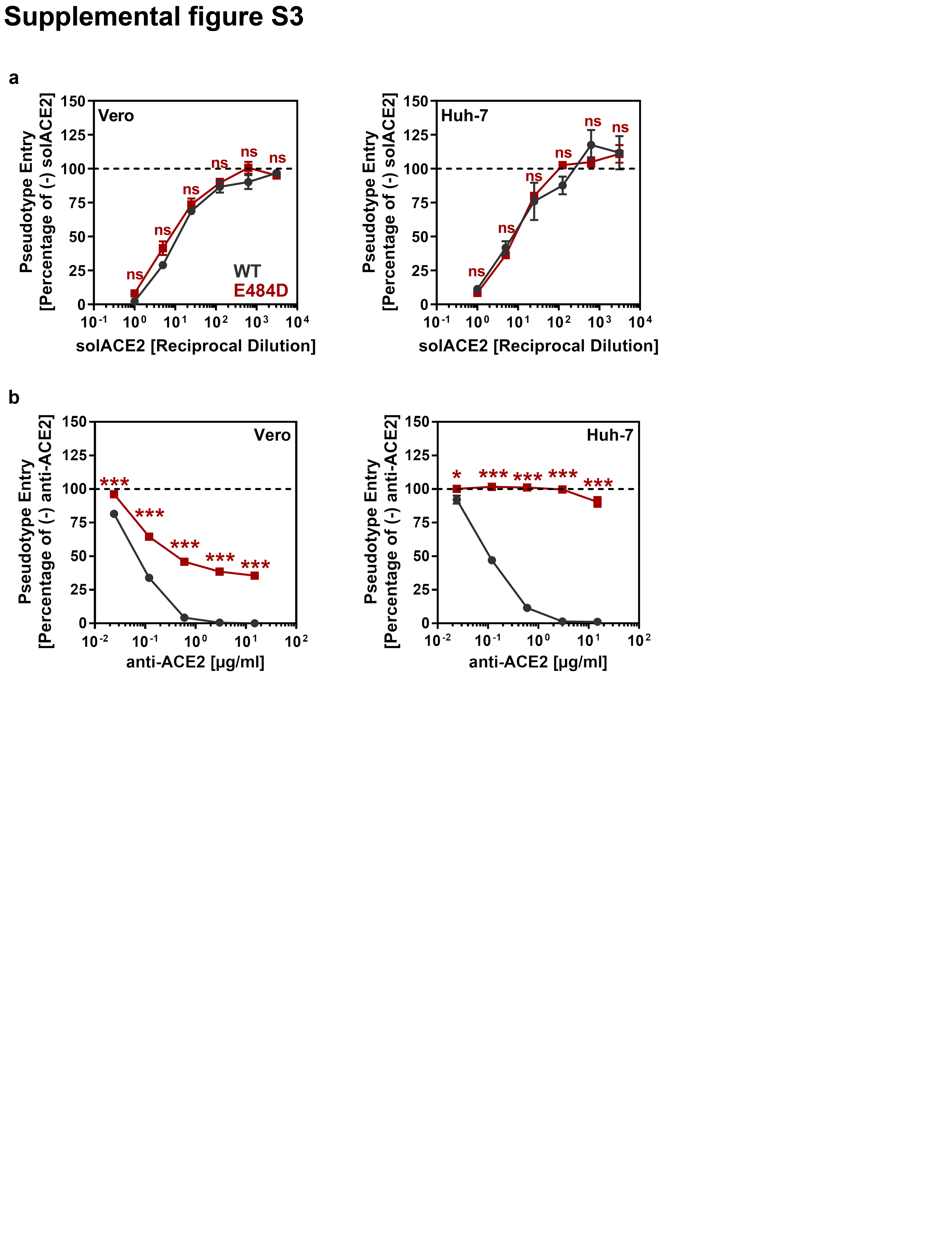

Supplement: FIG S3 [file mbio.00364-22-sf003.tif]

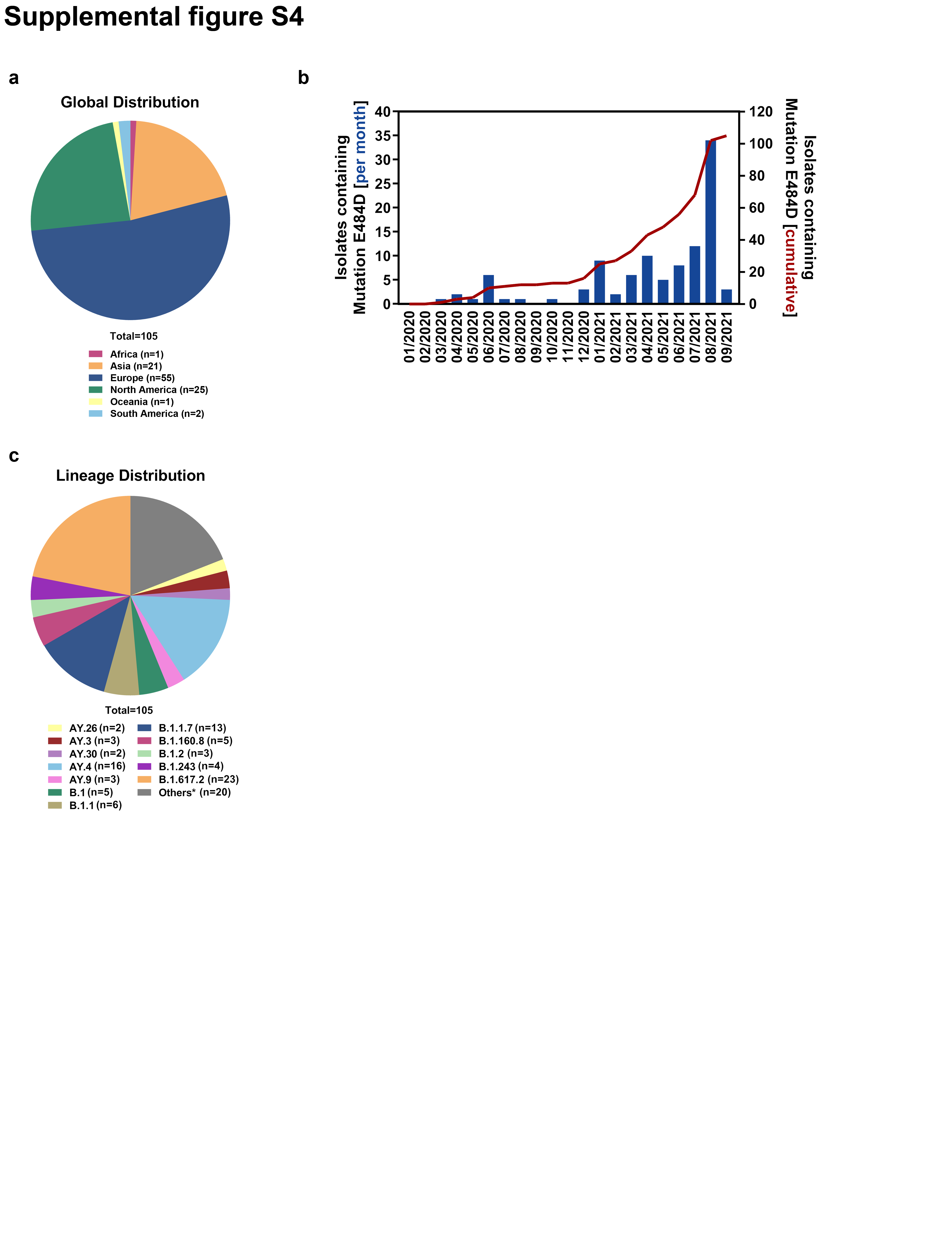

Supplement: FIG S4 [file mbio.00364-22-sf004.tif]

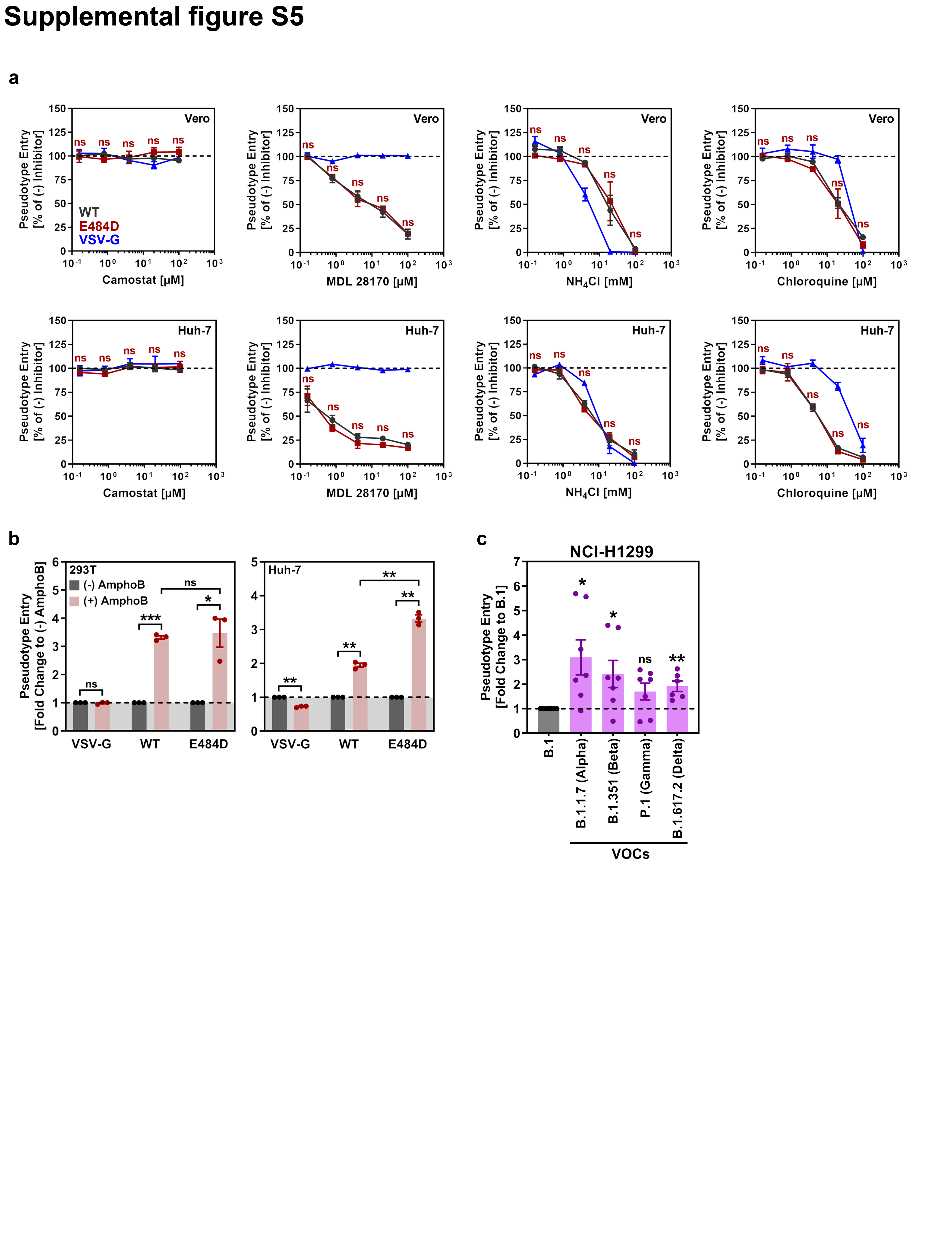

Supplement: FIG S5 [file mbio.00364-22-sf005.tif]
