# Supplementary material for: Evidence for an ACE2-Independent Entry Pathway That Can Protect from Neutralization by an Antibody Used for COVID-19 Therapy
Source: mBio. 2022 Apr 25;13(3):e00364-22. doi: 10.1128/mbio.00364-22 (PMC9239067; doi:10.1128/mbio.00364-22)
Supplement: TEXT S1 [file mbio.00364-22-s0001.docx]

**Materials and Methods**

**Cell cultures**

All cell lines were incubated at 37 °C and 5% CO_2_ in a humidified atmosphere. 293T (human, female, kidney; ACC-635, DSMZ, RRID:CVCL_0063), A549 (human, male, lung; CRM-CCL-185, ATCC, RRID:CVCL_0023; kindly provided by Georg Herrler), Huh-7 (human, male, liver; JCRB0403, JRCB; RRID:CVCL_0336, kindly provided by Thomas Pietschmann), HOS (human, female, bone; CRL-1543, ATCC; RRID:CVCL_0312) and Vero (African green monkey, female, kidney; CRL-1586, ATCC; RRID:CVCL_0574, kindly provided by Andrea Maisner) cells were maintained in Dulbecco´s´ modified Eagle medium (DMEM, PAN-Biotech). BEAS-2B (human, male, bronchus; CRL-9609, ATCC; RRID:CVCL_0168) and NCI-H727 (human, female, lung; CRL-5815, ATCC; RRID:CVCL_1584) cells were cultured in DMEM/F-12 medium (Thermo Fisher Scientific). Caco-2 (human, male, colon; HTB-37, ATCC, RRID:CVCL_0025) and Calu-3 (human, male, lung; HTB-55, ATCC; RRID:CVCL_0609, kindly provided by Stephan Ludwig) cells were maintained in Minimum Essential Medium (MEM, Thermo Fisher Scientific) and RPMI-1640 medium (PAN-Biotech) was used to culture NCI-H1299 (human, male, lung; CRL-5803, ATCC; RRID:CVCL_0060) cells. All media were supplemented with 10% fetal bovine serum (FCS, Biochrom), 100 U/ml of penicillin and 0.1 mg/ml of streptomycin (PAN-Biotech). In addition, Caco-2, Calu-3 and NCI-H1299 were grown in the presence of 10 mM sodium pyruvate (Thermo Fisher Scientific) and 1x non-essential amino acid mix (prepared from 10x stock, PAA). For cell line authentication, the following strategies were employed: Cell lines were validated by STR-typing, amplification and sequencing of a fragment of the cytochrome c oxidase gene, microscopic examination and/or according to their growth characteristics. Furthermore, cell lines were routinely tested for contamination by mycoplasma.

**Plasmids**

Expression plasmids for vesicular stomatitis (VSV) glycoprotein (VSV-G), soluble human ACE2 (equipped with a C-terminal Fc-tag, derived from human immunoglobulin G [IgG], sol-ACE2-Fc) and the spike (S) proteins of wildtype (WT) SARS-CoV-2 (Pango lineage B.1, differs from S protein of the Wuhan/Hu-1/2019 isolate only by mutation D614G; codon optimized, C-terminal truncation of the last 18 amino acid) or SARS-CoV-2 variants of concern (VOC) Alpha (B.1.1.7), Beta (B.1.351), Gamma (P.1) and Delta (B.1.617.2) have been described elsewhere.(1-4) Empty pCG1 expression plasmid was kindly provided by Roberto Cattaneo, Mayo Clinic College of Medicine, Rochester, MN, USA). Expression plasmids for SARS-CoV-2 S protein harboring amino acid exchanges at amino acid position 484 (E484A, E484D, E484G, E484K, E484Q) where generated by overlap extension PCR using the expression plasmid for WT SARS-CoV S as template. The resulting PCR products were cloned into the pCG1 plasmid making use of BamHI and XbaI restriction sites (Specific details on the cloning procedure will be provided upon request). The integrity of all sequences was confirmed by sequence analysis using a commercial sequencing service (Microsynth SeqLab). Transfection of 293T cells was achieved employing the calcium-phosphate precipitation method.

**Sequence analysis and protein models**

Information of the SARS-CoV-2 isolates harboring mutations at position 484 in the S protein were retrieved from the GISAID global initiative on sharing all influenza data) database (<https://www.gisaid.org/>). The trimeric S protein model was constructed by modelling the SARS-2 S sequence on PDB: 6XR8,(5) using the SWISS-MODEL online tool (<https://swissmodel.expasy.org>). In addition, the following protein structures for complexes of the SARS-CoV-2 S protein receptor binding domain (RBD) bound by monoclonal antibodies were used: PDB: 6XDG,(6) PDB: 7L3N(7) or PDB: 7C01.(8) Editing (e.g. coloring) of protein models was done using the YASARA software (<http://www.yasara.org/index.html>).

**Production of soluble ACE2 and analysis of ACE2 binding**

Soluble ACE2 was produced as described before.(3) In brief, 293T cells were transfected with sol-ACE2-Fc expression plasmid. At 10 h posttransfection, the transfection medium was aspirated and cells were further incubated. At 48 h posttransfection, the medium was collected and centrifuged (2,000 x g, 10 min, 4°C), before the clarified supernatant was loaded onto Vivaspin protein concentrator columns (30 kDa molecular weight cut-off, Sartorius). Next, the sample was centrifuged at 4,000 x g (4 °C) until a concentration factor of 20 was achieved. Concentrated soluble ACE2 was aliquoted and stored at -80°C until further use.

Binding of soluble ACE2 to SARS-CoV-2 S protein was investigated by flow cytometry. For this, 293T cells expressing WT or mutant SARS-CoV-2 S (or no S protein, control) upon transfection were washed with PBS and subsequently resuspended in FACS buffer (PBS containing 1% bovine serum albumin) at 48 h posttransfection. Following pelleting of the cells by centrifugation (600 x g, 5 min, RT; identical conditions for all subsequent centrifugation steps), the cell pellet was resuspended in FACS buffer containing soluble ACE2-Fc (1:100) and rotated for 1 h in a Rotospin eppi rotator disk (IKA) at 4 °C. Thereafter, cells were pelleted, washed with FACS buffer and pellet again, before being resuspended in FACS buffer containing AlexaFluor-488-conjugated anti-human antibody (1:200; Thermo Fisher Scientific). Samples were rotated again for 1 h at 4 °C. Then, cells were pelleted, washed with FACS buffer, pellet again and resuspended in 4 % paraformaldehyde solution (Carl Roth) for fixation (30 min, RT). Finally, cells were pelleted, washed with FACS buffer, pellet again and resuspended in FACS buffer, before being analyzed using an LSR II flow cytometer with FACS Diva software (BD Biosciences). For subsequent data analysis, FCS express 4 Flow research software (De Novo Software) was used.

**Analysis of binding of monoclonal antibodies to wildtype and mutant SARS-CoV-2 S**

293T cells were transfected with expression plasmids for WT or mutant SARS-CoV-2 S proteins or empty expression vector by calcium phosphate precipitation. Medium was replaced at 16 h post transfection and cells were further incubated for 32 h. Next, cells were washed with PBS, resuspended in FACS buffer and for each sample identical volumes were transferred into six separate reaction tubes. Cells were further pelleted by centrifugation (600 x g, 5 min, RT). After aspiration of the supernatant, for each sample one cell pellet was resuspended in either FACS buffer containing no monoclonal antibody (2nd Ab control) or FACS buffer containing either an unrelated human control antibody (hIgG), Casirivimab, Imdevimab, Bamlanivimab or Etesevimab (concentration: 1 µg/ml). Samples were mixed by vortexing and rotated for 1 h at 4 °C. Subsequently, samples were washed once with FACS buffer and further resuspended in FACS buffer containing Alexa Flour-488-conjugated anti-human antibody (Thermo Fisher Scientific; 1:250) and again rotated for 1h at 4 °C. Thereafter, samples were washed once with FACS buffer, fixed by incubation with 4 % paraformaldehyde solution (Carl Roth; 30 min, RT), washed again and analyzed using an LSR II flow cytometer with FACS Diva software (BD Biosciences). For subsequent data analysis, Flowing Software (version 2.5.1, Turku Bioscience) was used.

**Production of rhabdoviral transduction vectors pseudotyped with SARS-CoV-2 S**

For the generation of rhabdoviral transduction vectors a replication-deficient vesicular stomatitis virus was employed, VSV∗ΔG-FLuc, that was kindly provided by Gert Zimmer.(9) The genome of VSV∗ΔG-FLuc lacks the genetic information for VSV-G but codes for two reporter proteins, enhanced green fluorescent protein and firefly luciferase (FLuc), instead. Pseudotyping of VSV was carried out according to published protocol.(10) In brief, 293T cells were transfected with expression plasmids encoding S protein, VSV-G or empty plasmid (control) 24 h in advance to inoculation with VSV∗ΔG-FLuc at a multiplicity of infection of 3. At 1 h postinoculation, cells were washed with phosphate-buffered saline (PBS) and further incubated with culture medium containing anti-VSV-G antibody (culture supernatant from I1-hybridoma cells; ATCC no. CRL-2700; except for cells expressing VSV-G, which received only medium). At 16-18 h postinoculation, the pseudovirus-containing medium was collected, centrifuged at 4,000 x g for 10 min and the clarified supernatant was aliquoted and stored at -80 °C until further use.

**Transduction of target cells**

At 24 h prior to transduction, target cells were seeded in 96-well plates. The following experimental designs were chosen: (i) For experiments assessing the cell line tropism of rhabdoviral transduction vectors pseudotyped with WT or mutant SARS-CoV-2 S, the culture medium was aspirated and equal volumes of pseudotype particles were inoculated on the cells. Transduction vectors bearing VSV-G or no viral surface protein served as specificity controls. (ii) In order to address the impact of antibody-mediated ACE2 blockade on S protein-driven cell entry, target cells were preincubated (30 min, 37 °C) with medium containing different concentrations of anti-ACE2 neutralizing mouse monoclonal antibody (10108-MM36 or 10108-MM37, Sino Biological), before equal volumes of pseudotype particles were added on top. Cells incubated with medium without antibody served as control (= 100 % cell entry). (iii) Blockade of SARS-CoV-2 S protein-driven cell entry by soluble ACE2 or heparin was analyzed by preincubated (30 min, 37 °C) of transduction vectors with medium containing different dilutions of soluble ACE2 or concentrations of heparin (Sigma-Aldrich), before inoculating them on target cells. Transduction vectors incubated with medium without soluble ACE2 or heparin served as controls (= 100 % cell entry). (iv) To investigate neutralization of S protein-driven cell entry by antibodies, rhabdoviral transduction vectors pseudotyped with WT or mutant SARS-CoV-2 S were preincubated (30 min, 37 °C) with medium containing different concentrations of monoclonal anti-SARS-CoV-2 antibody (Casirivimab, Imdevimab, Bamlanivimab, Etesevimab) or unrelated human control antibody (hIgG), or different dilutions of previously described plasma from convalescent COVID-19 patients (n = 10,(11)), before being inoculated on target cells. Transduction vectors incubated with medium containing no antibody or convalescent plasma served as control (= 100 % cell entry). (v) To analyze restriction of S protein-driven entry by interferon induced transmembrane (IFITM) proteins, target cells were preincubated (1 h at 37 °C) in the presence 2.5 µM of the antifungal amphotericin B to block the antiviral activity of IFITM proteins, before pseudotype particles were added. Cells incubated in the absence of amphotericin B served as reference. (vi) In order to assess whether SARS-CoV 2 S-driven entry into Huh-7 cells depends on the activity of cathepsin L (membrane fusion in endosomes/lysosomes) or TMPRSS2 (fusion at the plasma membrane), target cells were preincubated for 1h at 37 °C in the presence of different concentrations of the TMPRSS2 inhibitor camostat mesylate (Sigma-Aldrich), the cathepsin L and B inhibitor MDL 28170 (Tocris), or the lysomotropic agents chloroquine (Tocris) and ammonium chloride (Sigma-Aldrich), before pseudotype particles were added. Cells incubated in the presence of diluent (water [ammonium chloride] or DMSO [camostat mesylate, MDL 28170, chloroquine]) served as reference.

For all transduction experiments, transduction efficiency was analyzed at 16-18 h postinoculation by measuring FLuc activity in cell lysates. For this, the culture medium was aspirated and cells were lysed by incubation (30 min, room temperature [RT]) with PBS containing 0.5% triton X-100 (Carl Roth). Lysates were subsequently transferred into white 96-well plates. Finally, FLuc substrate (Beetle- Juice, PJK GmbH) was added and luminescence was measured using a Hidex Sense plate luminometer (Hidex).

**Data analysis**

Results on S protein-specific differences in cell tropism represent average (mean) data obtained from three biological replicates (each performed with technical quadruplicates), for which transduction was normalized against WT S protein (set as 100 %). Alternatively, transduction was normalized against the (background) signals obtained from cells that had been inoculated with particles bearing no viral glycoprotein (set as 1). Results on binding of soluble ACE2 or monoclonal antibodies to WT and mutant SARS-CoV-2 S protein represent average (mean) data obtained from three biological replicates (each performed with single samples). Each data point represents the geometric mean channel fluorescence for one biological replicate without normalization. In addition, to directly compare antibody binding to WT and mutant SARS-CoV-2 S proteins, data were further normalized (binding to WT SARS-CoV-2 S was set as 100%). Results on inhibition of S protein-driven cell entry through antibodies against ACE2, soluble ACE2, heparin, or protease inhibitors (camostat mesylate, MDL 28170) and lysomotropic agents (chloroquine, ammonium chloride) represent average (mean) data obtained from three biological replicates (each performed with technical quadruplicates), for which transduction in the absence of anti-ACE2 antibody, soluble ACE2, heparin, protease inhibitor or lysomotropic agent was set as 100 %. Results on neutralization of S protein-driven cell entry by monoclonal antibodies represent average (mean) data that were obtained from a single biological replicate (performed with technical quadruplicates) and the results were confirmed in a separate experiment. For data normalization, transduction in the absence of monoclonal antibody was set as 0 % inhibition. Results on neutralization of S protein-driven cell entry by convalescent plasma represent averages (median), quartiles (25 % and 75 %) and ranges that were obtained from a single biological replicate (performed with technical quadruplicates). For data normalization, transduction in the absence of monoclonal antibody was set as 0 % inhibition and the neutralizing titer 50 (NT50) value (= plasma dilution that leads to 50 % reduction of transduction) was calculated using a non-linear regression model (inhibitor vs. normalized response, variable slope). NT50 values were further used to calculate the x-fold changes in neutralization sensitivity between particles bearing WT or mutant S protein, or between different cell lines. Results on augmentation of S protein-driven cell entry following preincubation of target cells with amphotericin B represent average (mean) data obtained from three biological replicates (each performed with technical quadruplicates), for which transduction in the absence of amphotericin B was set as 1.

Error bars indicate either the standard deviation (SD) or the standard error of the mean (SEM) and the type of error bars used is specified in the figure legends. Data ware analyzed using Microsoft Excel (as part of the Microsoft Office software package, version 2019, Microsoft Corporation) and GraphPad Prism 8 version 8.4.3 (GraphPad Software). Statistical significance was tested by two-tailed Students t-test with Welch’s correction, two-way analysis of variance (ANOVA) with Sidak’s post-hoc test, or two-tailed Mann-Whitney test (details on the statistical test are given in the figure legends). Only p values of 0.05 or lower were considered statistically significant (p > 0.05, not significant [ns]; p ≤ 0.05, *; p ≤ 0.01, **; p ≤ 0.001, ***).

**REFERENCES**

1. Arora P, Kempf A, Nehlmeier I*, et al.* 2021. Increased lung cell entry of B.1.617.2 and evasion of antibodies induced by infection and BNT162b2 vaccination. bioRxiv doi:10.1101/2021.06.23.449568:2021.06.23.449568.

2. Brinkmann C, Hoffmann M, Lubke A*, et al.* 2017. The glycoprotein of vesicular stomatitis virus promotes release of virus-like particles from tetherin-positive cells. PLoS One 12:e0189073.

3. Hoffmann M, Arora P, Gross R*, et al.* 2021. SARS-CoV-2 variants B.1.351 and P.1 escape from neutralizing antibodies. Cell 184:2384-2393 e12.

4. Hoffmann M, Zhang L, Kruger N*, et al.* 2021. SARS-CoV-2 mutations acquired in mink reduce antibody-mediated neutralization. Cell Rep 35:109017.

5. Cai Y, Zhang J, Xiao T*, et al.* 2020. Distinct conformational states of SARS-CoV-2 spike protein. Science 369:1586-1592.

6. Hansen J, Baum A, Pascal KE*, et al.* 2020. Studies in humanized mice and convalescent humans yield a SARS-CoV-2 antibody cocktail. Science 369:1010-1014.

7. Jones BE, Brown-Augsburger PL, Corbett KS*, et al.* 2020. LY-CoV555, a rapidly isolated potent neutralizing antibody, provides protection in a non-human primate model of SARS-CoV-2 infection. bioRxiv doi:10.1101/2020.09.30.318972.

8. Shi R, Shan C, Duan X*, et al.* 2020. A human neutralizing antibody targets the receptor-binding site of SARS-CoV-2. Nature 584:120-124.

9. Berger Rentsch M, Zimmer G. 2011. A vesicular stomatitis virus replicon-based bioassay for the rapid and sensitive determination of multi-species type I interferon. PLoS One 6:e25858.

10. Kleine-Weber H, Elzayat MT, Wang L*, et al.* 2019. Mutations in the Spike Protein of Middle East Respiratory Syndrome Coronavirus Transmitted in Korea Increase Resistance to Antibody-Mediated Neutralization. J Virol 93.

11. Hoffmann M, Hofmann-Winkler H, Kruger N*, et al.* 2021. SARS-CoV-2 variant B.1.617 is resistant to bamlanivimab and evades antibodies induced by infection and vaccination. Cell Rep 36:109415.
